# A Novel Method to Inspect 3D Ball Joint Socket Products Using 2D Convolutional Neural Network with Spatial and Channel Attention

**DOI:** 10.3390/s22114192

**Published:** 2022-05-31

**Authors:** Bekhzod Mustafaev, Anvarjon Tursunov, Sungwon Kim, Eungsoo Kim

**Affiliations:** 1 ICT Creative Convergence Department, Busan University of Foreign Studies, Busan 46234, Korea; bekhzodmustafaev91@gmail.com; 2 Department of Software, Sejong University, Seoul 05006, Korea; tursunovanvarjon@gmail.com; 3 Vazil Company Co., Ltd., Busan 46918, Korea; swk@vazilcompany.com; 4 Department of Artificial Intelligence Convergence Engineering, Busan University of Foreign Studies, Busan 46234, Korea

**Keywords:** quality inspection, attention mechanism, convolutional neural networks, deep learning

## Abstract

Product defect inspections are extremely important for industrial manufacturing processes. It is necessary to develop a special inspection system for each industrial product due to their complexity and diversity. Even though high-precision 3D cameras are usually used to acquire data to inspect 3D objects, it is hard to use them in real-time defect inspection systems due to their high price and long processing time. To address these problems, we propose a product inspection system that uses five 2D cameras to capture all inspection parts of the product and a deep learning-based 2D convolutional neural network (CNN) with spatial and channel attention (SCA) mechanisms to efficiently inspect 3D ball joint socket products. Channel attention (CA) in our model detects the most relevant feature maps while spatial attention (SA) finds the most important regions in the extracted feature map of the target. To build the final SCA feature vector, we concatenated the learned feature vectors of CA and SA because they complement each other. Thus, our proposed CNN with SCA provides high inspection accuracy as well as it having the potential to detect small defects of the product. Our proposed model achieved 98% classification accuracy in the experiments and proved its efficiency on product inspection in real-time.

## 1. Introduction

A ball joint socket is a sort of mechanical connection that is commonly seen in cars and other types of machinery. This connection works similarly to the ball-and-joint socket in the human hip. It is made up of a ball or bearing that swivels inside a socket. The socket is tiny enough to keep the ball in place and prevent it from falling out, but large enough to allow the ball to swivel or rotate as needed [1,2]. In many mechanical applications, the ball socket joint combines strength, flexibility, and shock absorption to help decrease wear. This type of socket joint is used by many modern car manufacturers to connect the car wheels to the axle. The ball joint socket allows the wheels to not only spin from side to side when driving, but also travel over bumps or potholes on the road. Without this joint, the automobile would suffer a lot of damage every time it struck a pothole or other tiny impediment. This joint serves to balance the car as the wheels collide with these impediments, reducing the driver’s stress. The ball joint socket protects the vehicle and prevents wear and tear on automotive parts by absorbing shock, thus extending the vehicle’s life [3].

Inspection of the product quality in real time is critical in manufacturing processes since an efficient quality monitoring system aids in preventing the cost and inconvenience of providing defective products to customers [4]. Many factories still use human laborers to inspect product quality. Even though human laborers inspect products with high precision because of their work experience and intelligence, there are some disadvantages that need to be solved. Usually, human inspectors are unable to perform the inspection process at a high level of precision during inspection time because of eye fatigue. They need to take a rest at certain times to work precisely. Moreover, more workers will be required if the production of the factory is increased, and each individual worker should have adequate experience of analyzing the products to ensure inspection quality. Due to the above factors, production efficiency and company reliability can be significantly reduced [5].

To overcome these problems, many studies have been conducted in vision-based systems in order to automatically identify defects in different types of products such as fabric, bottles, car body surfaces, and metals [6,7,8,9,10]. When compared to a human inspector, these kinds of automatic defect inspection systems have numerous benefits, such as continuous operation over a long period, high inspection accuracy results during the inspection time, and operation in a complex environment. However, there are many industrial products that vary in terms of product material, shape, and the varying sizes of defects. Due to the complexity and diversity of industrial products, each product in a factory requires the development of a special inspection system. Defects in flattened surface products would normally be identified on the product’s surface using 2D cameras. However, the inspection procedure for three-dimensional (3D) mechanical products, such as ball joint sockets, is substantially more challenging, because defects can occur in any part of the 3D products. In order to inspect all parts of the 3D mechanical product, 3D cameras are typically used to collect data for the inspection process [11]. Though, this kind of 3D mechanical product inspection systems impose some limitations when it is applied to a real-world manufacturing process. At first, 3D industrial cameras are very expensive compared to 2D industrial cameras, which raises the entire cost of the inspection system. Furthermore, dealing with 3D data is substantially more difficult, necessitating multiple preprocessing steps, and data processing time is also long, resulting in a major reduction in product manufacturing operations [12]. Thus, to overcome these issues we proposed a novel method to inspect 3D ball joint socket products using 2D cameras in our system.

In this study, we proposed a novel method to inspect a 3D ball joint socket which uses five 2D industrial cameras, to acquire all inspection parts of the product and convolutional neural network (CNN) with spatial and channel attention (SCA) to detect defects of the product efficiently and accurately. We primarily focused on 3D-shaped object defect inspection problems and detecting very small defects that could occur in any part of the object. The dataset was collected in a factory environment using five 2D cameras installed on a 3D ball joint socket inspection system. Since the socket product shape is 3D, we used five 2D industrial cameras to capture all parts of the object and inspected each part individually. Our proposed technique ensures high-speed and real-time performance on the inspection time of the product by using 2D industrial cameras. A light reflection problem occurred during the image acquisition. That is because the product material is metal, and the light reflection value of its surface was varied. We used a gamma correction algorithm to deal with this problem. To efficiently process acquired images we proposed a CNN model consisting of a feature extraction block (FEB) followed by SCA and two fully connected layers (FCN). Our proposed CNN with SCA provides high inspection accuracy as well as being robust to detect small defects of the product. Our proposed system provides the following major contributions:

- We developed, tested, and deployed a defect inspection system for efficient classification of defective and non-defective 3D ball joint socket products using a 2D convolutional neural network (CNN) with channel and spatial attention mechanisms.
- We proposed a novel method to inspect 3D objects using 2D cameras. Our system uses five industrial cameras to capture all inspection parts of the 3D ball joint socket product. Acquired 2D images are processed to detect defect products using 2D CNN with channel and spatial attention.
- Some parts of the product reflect light more than other parts and it leads to different light reflection issue. To solve this problem, first we reduced the light intensity to a certain value to make sure that there is no light reflection from the shiny parts of the product. Thereafter, we applied a gamma correction method to increase the brightness of the acquired image.
- We proposed a convolutional neural network with channel and spatial attention for accurate detection of defects of the 3D ball joint socket product. Our proposed CNN model with channel and spatial attention extracts useful features from the input image using a feature extraction block (FEB), and then channel attention detects the most relevant feature maps for the target while spatial attention finds the most important regions in the extracted feature map for the target. Both attention features are combined to make robust feature maps and are fed to the fully connected network to process and make final prediction.
- We experimentally proved the effectiveness of the proposed CNN model with channel and spatial attention by developing and analyzing the results of four different CNN models. Our proposed model achieved 98% average accuracy rate which was 18% higher compared to the CNN model without channel and spatial attention mechanisms.

The rest of this article is organized as follows: Section 2 contains an overview of the literature on vision-based inspection methods for automatically detecting defects in various products. Section 3 provides a comprehensive explanation of the proposed framework. Section 4 contains the data collection and dataset information. Experimental results are given in Section 5. The discussion and comparative analysis of the proposed system and obtained results is included in Section 6. Finally, Section 7 presents the conclusions and future directions.

## 2. Literature Review

### 2.1. Handcrafted Features with Traditional Machine Learning Methods

Over the past decades, there have been many studies in surface defect detection using traditional image processing and machine learning methods. Various machine vision algorithms are used for a defect inspection system, such as texture feature-based, color feature-based, and shape feature-based [13]. Text feature can represent the organization structure and arrangement properties of the image surface through the gray distribution of the pixels and their nearby spatial neighborhoods. A statistical method can be used to detect defects from the texture features of the image. The main idea of statistical methods is to use the gray value distribution on the surface of an object as a random distribution and use the statistics of the random variable through the local binary pattern. In [14], steel plate surface defects were detected using a multiblock local binary pattern algorithm with over 94% recognition accuracy. Color histogram features are also used to inspect defects of products. It describes the proportion of different colors in the entire image. Study [15] proposed a wood surface classification method based on the percentage color histogram feature and texture feature of the image block. Another popular defect inspection method is shape features of the image. The contour-based method is one of the most used shape features and it obtains the shape parameters of the image by describing the outer boundary feature of the object. Wang J. et al. [8] used Hough transform to extract a region of interest (ROI) from the input images and then CNN was used to inspect bottle products. Hough transform reduced the computation time by removing unrelated background in the input images. Multiple thresholds are often required with traditional methods to target defects in the algorithms, which are highly impacted by conditions such as lighting and background colors. The thresholds may need to be adjusted when a new problem arises, or the algorithms may even need to be redesigned. Additionally, handcrafted, or shallow learning techniques fail to distinguish a complex condition effectively. A specific scenario is used for these methods, meaning they are not very adaptable and robust.

### 2.2. Deep Learning-Based Methods

Modern approaches make use of a deep learning technique to achieve more reliable and efficient inspection performance in more complex scenarios. In recent years, several defect inspection methods based on deep learning have been proposed. A generic deep learning-based technique for the automated surface inspection (ASI) system was presented by Ren et al. [16], to inspect texture, color, and microstructure defects on different public datasets. The proposed ASI system involves image classification and defect segmentation tasks. First, images were divided into image patches then a pretrained convolutional neural network (CNN) was used to classify image patches into normal and defective categories. For each defect type, a heatmap was generated and a thresholding method was used for segmentation. The proposed ASI achieved a good result on both classification and segmentation tasks by improving the accuracy by 0.66–25.50% and 2.29–9.86%, respectively.

Aluminum casting product defect detection from X-ray images was proposed in [17]. They divided an X-ray image into several image patches and trained CNN model to classify image patches into defect and non-defect classes. In order to detect defect regions, first they used a sliding window method to process entire X-ray image and for each window they obtained defect or non-defect prediction from the trained CNN model. Afterwards, an image processing method was used to determine the defect region among all the sliding window results. Moreover, they used a generative adversarial network (GAN) and 3D ellipsoidal model to generate augmented defect samples. Their proposed model achieved 0.71 mAP on aluminum casting defect detection. Another study [4] proposed conditional convolutional variational autoencoders (CCVAE) to generate defect samples and deep CNN for metal surface defect inspection. They increased the performance of the deep CNN by 3.42% via training classification model on generated defect samples using CCVAE. Zheng X et al. [18] proposed a generic semi-supervised deep learning-based approach for ASI. Their proposed method uses MixMatch rules to augment defective data samples and a CNN based on residual structure to detect defects. The MixMatch [19] method generates new data samples by mixing images with labels and unlabeled images through the MixUp [20] method after data augmentation, label guessing, averaging, and sharpening. They achieved various accuracy results by applying different percentage of labeled training samples. Classification accuracy of 99.83% was achieved when 70% of labeled training samples was used for the model.

### 2.3. Handcrafted Features with Manifold-Valued Neural Network

A novel deep neural network (DNN) framework called ManifoldNet was proposed in [21]. An objective deep network to be regarded as a generalization of CNN to manifold-valued inputs through purely intrinsic operations on the manifold. They consider this as a potential analog of a CNN for handling images that are manifold-valued, that the value set is on a Riemannian manifold [22]. A number of experimental results demonstrate the effectiveness of ManifoldNet for computer vision and medical imaging applications [23,24], such as, a classification of diffusion tensor images from Parkinson disease patients and controls were trained and tested using a dataset containing diffusion weighted magnetic resonance (MR) images acquired at the University of Florida for 355 subjects with Parkinson’s disease (PD) and 356 controls (healthy). For this experiment two different ManifoldNet based approaches are used along with a traditional CNN model. In the first approach, diffusion tensors are used to capture the local diffusion process within a voxel utilizing symmetric positive definite matrix [25] and a second approach of representing orientation distribution functions (ODFs) is through the ensemble average of the probability density function [26]. Test results for the first and second approaches of ManifoldNet achieved around 95% and 94% accuracy, respectively, and both are higher than Resnet-34 CNN model which achieved about 71% accuracy.

Even though many deep learning methods have been developed by researchers, defect inspection of industrial products requires specific inspection methods depending on factors such as product material, product shape complexity, and defect types. When the product material is shiny metal, light reflection will be the first issue in developing the product inspection system. Detecting defects of various types, such as small or tiny defects, is challenging. Moreover, product shape complexity is one of the main factors to consider in choosing the right algorithm for product inspection.

## 3. Proposed Method

**Figure 1:**
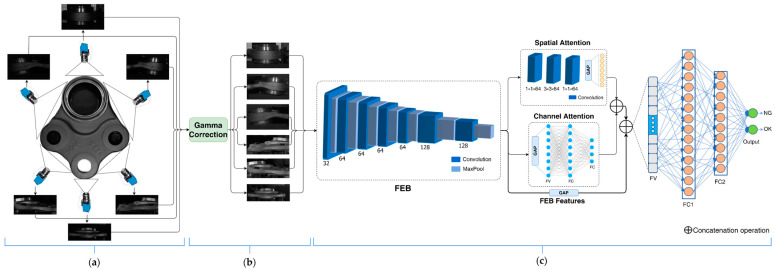
An overview of the proposed defect inspection framework. (**a**) Inspection surface acquisition, (**b**) image preprocessing, (**c**) proposed CNN model with the spatial and channel attention (SCA). FEB—feature extraction block. GAP—global average pooling, FV—feature vector, FC—fully connected layer.

In this section, we explain the proposed framework for the inspection of 3D ball joint socket products using a 2D convolutional neural network (CNN) with spatial and channel attention (SCA). Figure 1 illustrates the general overview of the proposed framework. We used five 2D cameras to capture the entire surface of the 3D ball joint socket. Gamma correction was used to fix the issue of different light reflection from the surface of the product. Our proposed framework utilizes 2D CNN with SCA for effective classification of defective and non-defective 3D ball joint socket products. The proposed model architecture consists of a feature extraction block (FEB), SCA, and fully connected network (FCN), which learns to classify the defective and non-defective products from the extracted feature vectors by previous blocks. A detailed explanation of the proposed framework components is presented in the upcoming sections.

### 3.1. Data Acquisition

Typically, building deep learning (DL) models requires sufficient data, as well as the data preparation process being one of the key factors of increasing the model accuracy and robustness. The improper organization of this process directly affects the model’s ability to learn and achieve high accuracy. Data collection and preprocessing require different approaches depending on the task and object properties such as the object shape, material type, etc. In the case of ball joint socket product inspection, the object has 3D shape and defects can occur in any part of the object during the manufacturing process. Generally, 3D cameras are used to acquire data for 3D shape objects and in order to process and accomplish different tasks such as binpicking and inspection. However, we used 2D cameras to inspect 3D ball joint sockets (Figure 1a). There are two primary intentions for using 2D industrial cameras to inspect the 3D shape object instead of a 3D camera. Firstly, high precision 3D industrial cameras are much expensive compared to 2D industrial cameras. Secondly, there is a significant difference between processing 3D data points, and 2D image data, which makes the usage of 3D cameras very difficult in fast real time processing.

### 3.2. Gamma Correction

Light reflection on the surface is always one of the most important problems that could impact on the system performance efficiency. Light reflection usually occurs on the surface of highly reflective metal objects. Recently, a gamma correction method was used to reduce the effect of light reflection on images [27]. The light intensity varies nonlinearly in the image captured by a camera. The gamma correction algorithm changes this nonlinearity by raising non-negative input values to the power of gamma value and multiplied by a certain constant value, and the output picture has the intended luminance. To fix the light reflection and enhance the image quality automatically, a gamma correction algorithm has been applied in this work. First, we reduced the light luminous intensity value of the light emitting diode (LED) light bar until the reflection in any part of the object disappeared, then images were acquired with that condition. The image brightness was then changed according to given gamma values (Figure 1b).

Assume that *P* denotes an interval of image pixel values between (0, 255), Ω means the angle value [0, π], *G* means the gamma value, and *k* represents the pixel value. Let *k_m_* be the middle point of *P*. Thus, the mapping from *P* to Ω is defined as:

(1) $$\begin{array}{c} \varphi :P\to \Omega ,\;\Omega =\{\omega |\omega =\varphi \left(k\right)\}\\ \varphi \left(k\right)=\pi k/2k_{m} \end{array}$$

The mapping from Ω to *G* is defined as:

(2) $$\begin{array}{c} 𝒽:\Omega \to G,\;G=\{\gamma |\gamma =𝒽\left(k\right)\}\\ \{\begin{array}{c} h\left(k\right)=1+f_{1}\left(k\right)\\ f_{1}\left(k\right)=a\;\cos(\varphi \left(k\right)) \end{array} \end{array}$$

where *a* ∈ (0, 1) is a weighted coefficient. Based on this map, there are related correlations between pixel value groups *P* and *G*. An arbitrary pixel value is related to a numerical gamma value that has been calculated. Let *γ*(*k*) *= h*(*k*) and choose gamma correction function as follows:

(3) $$g\left(k\right)=255{\left(k/255\right)}^{1/\gamma \left(k\right)}$$

where *g*(*k*) represents the output of correction value for pixel grayscale *k*. In this manner, the correction value of pixels is connected to the original pixel values, which achieved the requirements of image correction.

### 3.3. Feature Extraction Block

In the field of computer vision, current state-of-the-art results are attained by utilizing CNNs to perform various tasks, such as image classification [28], image segmentation [29], and object detection [30]. Usually, convolution layers, pooling layers, and fully connected layers are the three main elements of a CNN model. Convolution is used to extract valuable information from an image. Normally, the initial convolution layer captures low-level information, such as edges, color, gradient orientation, and with additional layers, architecture adjusts to high-level features as well, giving us a network with a comprehensive grasp of the pictures in the dataset. The pooling layer is responsible for lowering the dimension of extracted feature maps. The pooling layer also reduces the model’s processing time. As the output of the convolutional layer represents non-linear combinations of high-level features, fully connected layers learn how to classify defective and non-defective products from high-level features.

Seven convolution (C) layers were used to create FEB, followed by the same number of layers of max-pooling layers. Table 1 provides details about our proposed model’s configuration. To obtain useful local patterns from the input image, we used 32 kernels with dimensions 3 × 3 and a stride of 1 × 1 in the first convolution (C1) layer. To improve the model’s performance and to generalize it during training, we used the rectified linear unit (ReLU) as an activation function in all the convolution layers. From the second to the fifth convolution (C2–C5) layers, there are 64 filters, each with a size of 3 × 3 and a stride of 1 × 1, in order to produce a feature map, which is input to the next layer. The sixth and last convolution (C6–C7) layers contain 128 filters with 3 × 3 size of kernels and a stride with 1 × 1, the padding set to SAME (In SAME padding, when stride = 1 the output size of the convolution is the same as the input size. The same padding works by appending zero values in the image outer frame, thus the filter can cover the edges of the matrix and include them in the inference as well) to extract deeply hidden cues from the input data.

The max-pooling layer with pool size and stride of 2 × 2 is applied after each convolution layer. In addition to reducing the feature map dimensions, max-pooling reduces the computation cost for the network.

### 3.4. Proposed Spatial and Channel Attention

A number of tasks, such as text classification, machine translation, and speech recognition [31,32,33], have demonstrated the value of utilizing attention mechanisms in deep learning models. Attention works by focusing on the extracted features to select essential features of the target. It is not always the case that all extracted features apply to a target in any deep learning task. CNNs are very good at extracting useful salient feature maps from the input image. The extracted high-level salient feature maps are used to differentiate the objects with high accuracy in the task of classification. However, it is challenging to capture very small defects using those extracted feature maps, because similarity of defective and non-defective images is very high. In order to achieve high performance, all the useful features of the input data must be captured. To address this problem and enhance the classification model’s performance and robustness, we proposed spatial and channel attention (SCA) mechanisms to pay attention to the special part of the extracted feature maps as well as to effectively capture the importance of each extracted feature map. Figure 1c illustrates the SCA precise architecture.

The channel attention module exploits which feature map is the most relevant among all feature maps generated by FEB. To accumulate the feature map in each channel, we first applied global average pooling to the feature map following two fully connected layers and produced a channel attention vector $AV_{C}\in R^{CH/r}$ where, *r* is reduction ratio. The hidden layers of the first and second fully connected layers is set to 128 and 64, respectively. The output vector size of the channel attention module is 64. The final channel attention vector is calculated as follows:

(4) $$AV_{C}\left(F\right)=FC_{2}(FC_{1}\left(GAP\left(F\right)\right))$$

where *F* indicates the feature map, *FC* denotes a fully connected layer, and GAP denotes the global average pooling.

The spatial attention module learns the important parts of the each extracted feature map during training. The spatial attention module has three convolution layers and a global average pooling layer which retains useful features in output feature vector. Three convolution operations are applied to the input feature maps of $F\in R^{W\times H\times CH}$. The first convolution layer contains 64 kernels with a size of 1 × 1, and the second convolution layer includes 64 filters with the size of 3 × 3, and finally, the last convolution layer has the same parameters as the first convolution layer. The output dimension of this attention module is the same as that of the channel attention module. The final spatial attention vector $AV_{S}\left(F\right)$ is computed as follows:

(5) $$AV_{S}\left(F\right)=GAP\left(Z_{3}^{1\times 1}\left(Z_{2}^{3\times 3}\left(Z_{1}^{1\times 1}\left(F\right)\right)\right)\right)$$

where *Z* denotes a convolution operation and the superscripts represent the sizes of the convolution filters.

After computing the attention vectors from two separate branches, which are focused on which features and where the most relevant part of the input feature maps is located. To build our final attention vector *AV*(*F*), we combined these two attention vectors. The attention values estimated at two distinct channel and spatial branches complement each other, because the extracted attention vectors are different even though the input feature map is the same for both branches. Channel attention extracts the most relevant feature maps while spatial attention finds the most important regions in the extracted feature map. To facilitate gradient flow, we used a residual learning method conjunction with the SCA. To compose an efficient and robust attention module, we first compute the channel attention vector $AV_{C}\left(F\right)\in R^{CH/r}$ and the spatial attention vector $AV_{S}\left(F\right)\in R^{CH/r}\;$values at two separate branches. The final attention vector is computed as follows:

(6) $$AV\left(F\right)=AV_{C}+AV_{S}$$

In order to combine computed attention vectors with extracted feature maps, we converted feature maps into feature vectors using GAP operation. At first, learned attention vectors $AV\left(F\right)\epsilon R^{CH/r}$ are computed from the given input feature map $F\in R^{H\times W\times CH}$ that is extracted by the FEB, while feature vector $FV\left(F\right)\epsilon R^{CH}\;$ is computed from the input feature map by going through GAP, and the final refined feature vector $FV^{'}$ prime is calculated as follows:

(7) $$FV^{'}=AV\left(F\right)+FV\left(F\right)$$

## 4. Data Collection and Dataset Information

### 4.1. Ball Joint Socket Product Inspection System

**Figure 2:**
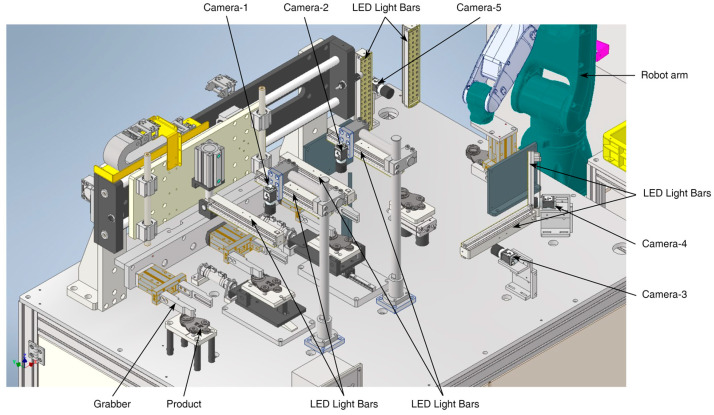
The 3D representation of the 3D ball joint socket defect inspection hardware system. LED—light-emitting diode.

The 3D ball joint socket defect inspection system contains a personal computer (PC) with a GPU device, five 2D industrial cameras, and a touch screen monitor. The defect inspection system is capable of detecting the defects of the product ranging from small to big sizes in real time in the product production process. The 3D model representation of the defect inspection system is shown in Figure 2. Alternatively, an inspection system for 3D ball joint sockets can be built in a different way. For instance, a robot arm grabs each product for inspection and then first two cameras take images of the front and back sides of the product. Then, a third camera takes side images of the product while the robot arm rotates the product horizontally. In this case, three 2D cameras would be enough. However, one of the main disadvantages of this system would be inspection time. Long inspection time comes from the two main aspects, which are: (a) the robot should change the grab point to inspect the sides of the product after completing the inspection of the front and the back sides of the product; and (b) during the product side inspection, the robot arm should rotate the product horizontally to some degree as well as it should move the product horizontally back and forth according to the side shape of the product towards the camera in order to adjust the camera focus to acquire high quality images.

According to the requirements of the project in question, the 3D ball joint socket inspection system must inspect 5000 products per day. Thus, the time limit for the inspection of each product must not exceed 6 s. To reduce the processing time of the inspection system, we built an inspection system that uses a sequential inspection method. We used five 2D cameras to inspect the entire surface of the products sequentially.

**Figure 3:**
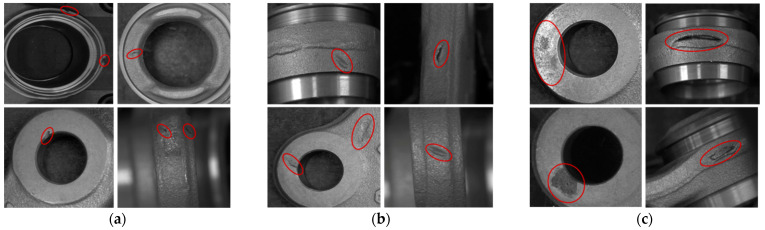
Demonstration of 3D ball joint socket product defects and defect sizes. (**a**) Small defects—1.5–3 mm, (**b**) medium defects—3–6 mm, (**c**) big defects—6–10 mm.

The whole process of our implemented inspection system begins when the product is inserted into the equipment. Before starting the image acquisition process, a grabber installed on top of the object placement location moves the product to the next stage camera for capturing. This process continues until the fourth camera finishes capturing the part of the product. Afterwards, the industrial robot grabs the object and moves to the fifth camera to complete the image acquisition process. Once the entire surface of the product is acquired from the 2D industrial cameras, the product classification process begins. Finally, the robot places the object onto the designated place based on the classification result. The resolution of the camera is 2592 × 1944 (height × width) pixels, which acquires high quality images. The first and second cameras acquire the top and bottom surfaces of the object. The third camera captures the front part of the product while the fourth camera acquires first the right front side of the product and then the left front side after rotating the product 90 degrees anticlockwise. Finally, the robot hand grabs the product and moves it to the fifth camera to capture the back side of the product, and then rotates it 90 degrees anticlockwise to capture the right back side, and 180 degrees clockwise to acquire the left back side of the product. When all acquired images from five 2D cameras are predicted as non-defective by our convolutional neural network (CNN) with spatial and channel attention (SCA), the robot arm puts the product into the good category, otherwise the product is considered as defective. An example of product defect is shown in Figure 3. Defect size is divided into three types, that is, small, medium, and large size defects are 1.5–3, 3–6, and 6–10 mm, respectively.

### 4.2. Dataset Description

To develop a robust defect inspection system utilizing deep learning (DL) algorithms, we must deal with a number of DL requirements. For example, the amount of training samples in each class should be a sufficient in order to learn and generalize the problem for the DL model. However, to solve computer vision challenges in industry, such as image classification and defect inspection, we must create our own dataset from scratch.

We obtained the data from the 3D ball joint socket product inspection system in a factory environment. Of the collected data, 80% was used to train a model and the remaining 20% of the data was used to test model performance. The training data contains 1014 non-defective and 734 defective samples. In the test data, there were 150 defective and 255 non-defective samples. A total of 2153 3D ball joint socket product images were used in the dataset. A detailed description of the dataset is given in Table 2.

## 5. Experiments and Results

### 5.1. Experimental Setup

We developed our proposed model using TensorFlow open-source framework [34], and the Python programming language [35]. The developed model was trained on an NVIDIA GeForce RTX 3090 GPU which has 24 GB of graphic memory. The detail specifications of software and hardware are listed in Table 3.

The 3D ball joint dataset is divided into 80% for training and 20% for testing. The training set was used to train our proposed convolutional neural network (CNN) with spatial and channel attention (SCA), the model and test set was used to verify the model performance after training. Training parameters were set as follows: input image dimension was 380 × 380 × 3 (height × width × channel), training iterations was set to 100, learning rate was set to 0.001, number of images in each batch was set to 128, and momentum was 0.99. The Adam optimization algorithm was used to optimize model parameters during training. The categorical cross entropy loss function was used to measure the prediction error of the model.

### 5.2. Evaluation of Model Testing Performance

Generally, classification model performance is measured using statistical metrics, such as confusion matrix, classification accuracy, precision, recall, and F1 score. Statistical metrics are computed from the model prediction and ground truth labels. A detailed descriptions of these metrics are described below.

True positive (TP) indicates the number of correctly predicted positive data samples. False positive (FP) indicates the data samples which the model predicted as positive but they belong to the negative class. True negative (TN) shows the number of samples for which model prediction and actual class labels are matched. False negative (FN) indicates that the predicted values of the model are negative, while actual class values of the samples are positive.

(8) $$\mathrm{Accuracy}=\frac{\mathrm{TP}+\;\mathrm{TN}\;}{\mathrm{TP}+\;\mathrm{FP}+\;\mathrm{FN}+\mathrm{TN}}$$

(9) $$\mathrm{Precision}=\frac{\mathrm{TP}\;}{\mathrm{TP}+\;\mathrm{FP}}$$

(10) $$\mathrm{Recall}=\frac{\mathrm{TP}}{\mathrm{TP}+\;\mathrm{FN}}$$

(11) $$F1-\mathrm{score}=\frac{2\times \mathrm{TP}}{2\times \mathrm{TN}+\;\mathrm{FP}+\mathrm{FN}}$$

Accuracy is the percentage of correctly classified objects. It can be defined as the ratio between the number of correctly predicted samples to the total number of samples. The accuracy factor is not enough to measure the performance and efficiency of a model. Hence, additional measurement factors are used, such as precision in Equation (9), which is a proportion of all correctly predicted positives to all predicted positives. Precision reflects how reliable the model is in identifying positive samples. Recall in Equation (10) indicates the proportion of all correctly predicted positives to all real positives. Recall measures the model’s ability to detect positive samples. F1-score in Equation (11) will give us the harmonic mean of precision and recall. We can think of it mathematically, F1 score is the weighted average of precision and recall.

### 5.3. Experimental Results

**Figure 4:**
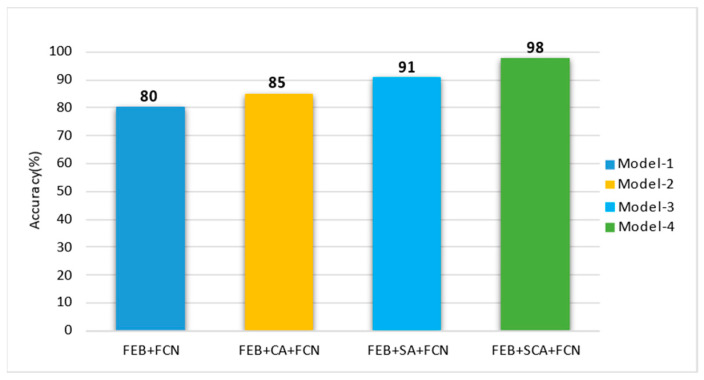
Classification performance comparison of our developed CNN models. All CNN models were trained to classify the 3D ball joint socket product into defective and non-defective categories.

Our proposed classification framework is practically evaluated using the test set of the 3D ball joint socket dataset to demonstrate the model efficiency and robustness. We developed four different CNN models in order to evaluate the proposed CNN with spatial and channel attention (SCA). We conducted experiments to classify the 3D ball joint socket product into defective and non-defective classes. The prediction performances of all four CNN models are shown in Figure 4.

The first CNN model (Figure 4, model-1) consists of the feature extraction block (FEB) and the fully connected network (FCN). This model used as the baseline model for analyzing the effectiveness of other CNN models together with attention modules. The second model (Figure 4, model-2) was built using FEB and channel attention (CA) followed by FCN. This model was developed to verify the effectiveness of the CA mechanism. The third model (Figure 4, model-3) has the same design as the second model, but CA has been replaced by spatial attention (SA). Finally, to determine if CA and SA features complement each other when combined, we developed the CNN model (Figure 4, model-4) using the FEB and SCA, followed by the FCN.

**Figure 5:**
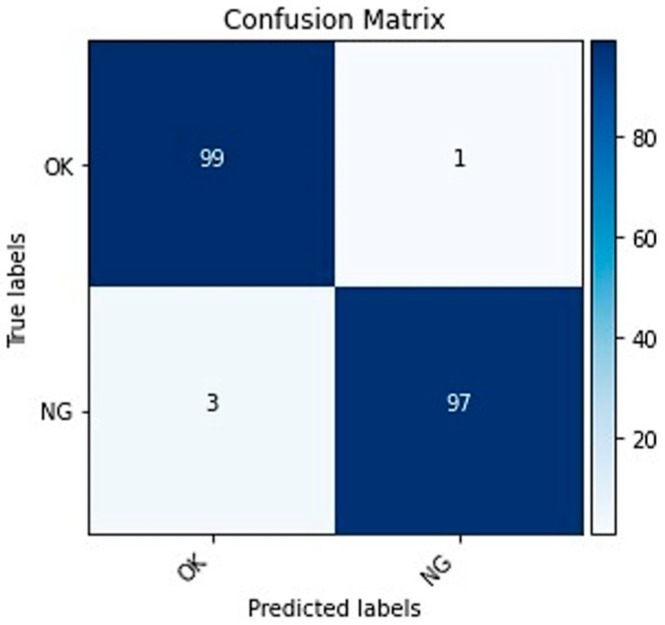
Confusion matrix of the proposed model for the 3D ball joint socket test dataset with 98% average accuracy score. OK and NG indicate the non-defective and defective samples, respectively.

The primary purpose of the last model is to analyze and represent the effectiveness of the SCA module when it is all combined. The output from the model are the class probabilities taken from the SoftMax layer. The final model prediction class was determined based on the class with the highest prediction probability. Following that, an accuracy score was computed using the final predicted class and the original labels. We can see from the results that SA features in model-3, provided better classification accuracy compared with CA features in model-2. This implies that SA features are more efficient than CA features in the task of 3D ball joint inspection. The results of model-4 indicated the efficiency of the SCA module for product classification using input images. The highest performance was achieved using the proposed model-4 among the other CNN models, which indicates that the combination of the SA and CA modules together with FEB and FCN in the CNN models is significant for obtaining high accuracy. The confusion matrix for classification of products into non-defective (OK) and defective (NG) categories is given in Figure 5. The results were obtained from the model predictions and the actual labels for the 3D ball joint socket test dataset. The best accuracy of 99% was achieved in the OK class and 97% classification accuracy was achieved for NG class. Table 4 presents the statistical factors obtained from the model prediction for defect classification using test data. The highest precision value achieved 98%, recall, and F1-score values achieved 99%, respectively. The average classification accuracy was achieved at 98%.

## 6. Discussion and Comparative Analysis

Defect inspection of industrial products requires specific inspection methods depending on factors such as product material, product shape complexity, and defect types. When the product material is shiny metal, light reflection will be the first issue in developing a product inspection system. Defect type, such as small or tiny defects, makes the defect inspection task challenging. Moreover, product shape complexity is one of the main factors to consider in choosing the right algorithm for product inspection. Various works have been conducted on surface inspection algorithms [36,37,38,39]. The majority of the developed inspection algorithms attempt to detect defects on the product’s surface. Furthermore, the shape of 3D industrial products is typically complex, creating a variety of challenges that require a unique approach to solve problems. To efficiently inspect the defects of 3D ball joint socket products, we developed a new method that uses five 2D cameras to capture all the inspection surfaces of the product as shown in Figure 2. There are two main advantages of the proposed method: (a) 2D cameras are significantly cheaper than 3D cameras. As a result, the cost for developing an inspection system will be reduced, (b) the processing time of 2D images is considerably short compared to 3D camera data. Consequently, the speed of the inspection system will be increased significantly. Inspection time in our system is six seconds for a single product using 2D industrial cameras.

**Figure 6:**
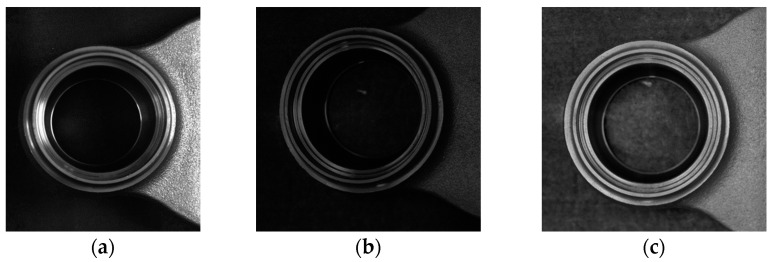
Illustration of light reflection issue of 3D ball joint socket object. (**a**) Light reflected, (**b**) before gamma correction, (**c**) gamma applied image.

Lighting effects of the captured image from the camera depend on factors such as outside lights source and product material. The material of the 3D ball joint socket is metal and some parts of the product have high light reflectance compared to other parts as shown in Figure 6. Thus, light reflection from the product will not be the same in all parts of the product. Therefore, balancing the light source and intensity will be the issue. When the intensity of the light source is high then light reflection from the shiny parts of the product will be high compared to less light-reflective parts. If the light source intensity is low, then capturing the less light-reflective parts of the product will be difficult. In both conditions, detection of the product defects will be very hard. To solve this issue, first we reduced the light intensity to a certain value to make sure that there is no light reflection from the shiny parts of the product. Second, we applied the gamma correction method in order to increase the brightness of the captured image. After applying gamma correction, all parts of the product will be clearly visible without any light reflection.

**Figure 7:**
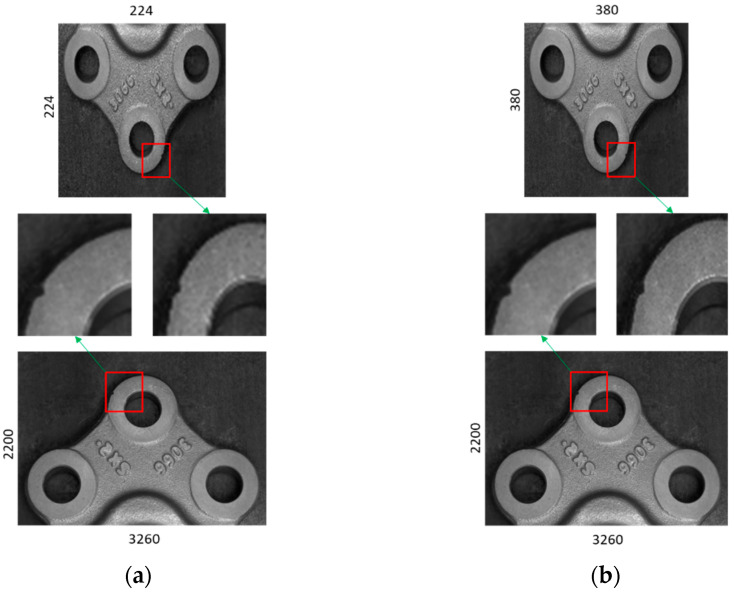
The impact of image size in small defects of the product in resizing of original image. (**a**) A resized image with 224 × 224 image size, (**b**) a resized image with 380 × 380 image size.

The performance of the product defect inspection system is reliant on various factors such as defect type and inspection algorithm. Small defects of the product (Figure 3a) make the defect inspection challenging. To efficiently detect small defects every step from the selection of good quality cameras to the final decision-making algorithm in the inspection process needs to be developed correctly. To inspect the 3D ball joint socket, five cameras capture different parts of the product with different size. The next step is resizing different sized images into the same size to process in the defect detection algorithm. Choosing the right size for the resized image is crucial to increase the performance of the inspection system. Figure 7 shows the resized image size impact on the small defect of the 3D ball joint socket. Resized image quality becomes noticeably poor when the difference between the original image and resized image size is quite big (Figure 7a). As a result, small defects of the product almost disappear in the resized image and make the defect detection task much harder. Two main factors were considered when choosing the right resized image size, namely, the recognition rate of the defect inspection algorithm and computation resources. Image size of 380 × 380 (height × width) (Figure 7b) was selected from the extensive experiments which maintains the balance between the model performance and computation resources.

For the purpose of accurately separating defect products from good quality products, we developed the convolutional neural network (CNN) consisting of convolutional, max pooling, and fully connected layers. Our CNN model without spatial and channel attention (SCA) (Figure 4 model-1) achieved 80% average accuracy rate on testing data. Even though this model detected medium and big sized defect types (Figure 3b,c), it cannot detect most of the small defects (Figure 3a) on the product, because this model processed all extracted feature maps with equal importance to the target. To fix this issue, we developed a CNN with channel attention (CA) mechanism (Figure 4, model-2). Channel attention is located between FEB and FCN. The main function of channel attention is learning the importance of each feature map to the target during training. Model-2 achieved 85% average accuracy rate which is 5% higher compared to model-1. Even though the CNN with CA detected small defects with higher accuracy than model-1, there was still misclassification of small defects of the product. That is because the CA selects only important feature maps among all extracted feature maps; however it does not pay attention to which areas of the selected feature maps are important. To increase the recognition rate of small defects, we developed a CNN with spatial attention (SA) mechanism (Figure 4 model-3), and CA was replaced with SA. One of the main reasons that SA improves the recognition rate of small defect types is that it learns to find the most important part of the extracted feature map to the target. Model-3 detected defects with 91% average accuracy rate which is 11% and 6% higher than model-1 and model-2, respectively. From the experiments, it was clear that both CA and SA increased the performance of the CNN. In order to know whether CA and SA complement each other or not, we developed the CNN model with SCA (Figure 4, model-4). Model-4 classified defect and good products with average 98% accuracy rate. Model-4 achieved 18% higher classification accuracy than model-1. Experiments proved that CA and SA mechanisms complemented each other and increased the detection rate of defect products. Model-4 was used in a 3D ball joint socket defect inspection system and deployed to the factory for automatically inspection of defective products.

**Figure 8:**
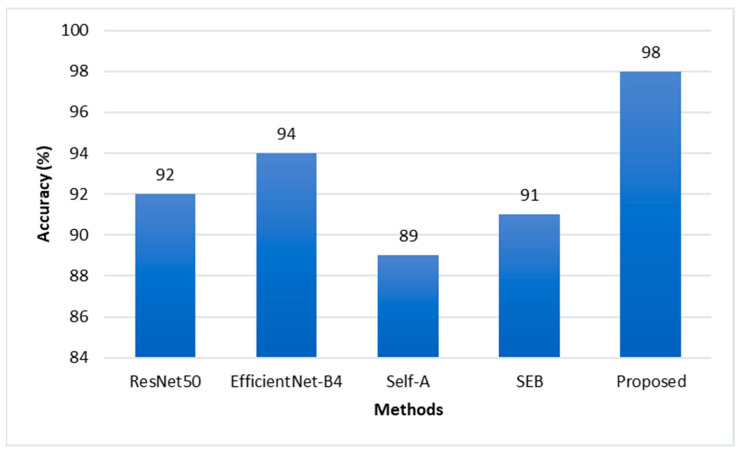
Proposed model performance comparison with existing methods. Squeeze-and-excitation networks (SEB) [40], self-attention generative adversarial networks (Self-A) [41].

Moreover, to analyze our model performance with other existing methods we compared our CNN model with SCA with two different attention mechanisms and pre-trained CNN models using the 3D ball joint socket dataset. The result of comparing the performance of the proposed model and other existed methods is shown in Figure 8.

Squeeze-and-excitation block (SEB) [40] squeezes the learned feature maps in order to gather global spatial information to form a channel descriptor. Global average pooling operation is used to obtain channel-wise statistics. Two fully connected layers are used to learn the hidden relationship between feature map channels and a sigmoid activation function is used to give the importance score for each feature map channels. These learned important scores are multiplied with the input feature map to form channel-wise attention feature maps. As a result, the network learns to give different importance scores for each extracted feature map instead of considering every feature channel with equal importance. Thus, this model gave 91% average classification accuracy which is 11% higher than our model-1 because of additional SEB attention. However, only SEB attention is not good enough to inspect defects with high precision because it only tries to learn channel-wise attention. It lacks capturing spatial importance.

The convolution operation processes the information in a local neighborhood because of the small receptive field. It lacks modeling long-range information in the images. The self-attention (Self-A) mechanism [41] helps the convolution operation to model long-range information by attending to all spatial regions of the input image. The self-attention module computes attention value at a specific location as a weighted sum of the spatial features at all positions. When the convolution operation is combined with the self-attention module, the neural network has the ability to process information not only with local receptive field but also paying attention globally. As a result, when we added Self-A block to our model-1 architecture, it recognized defect products with 9% higher accuracy rate compared to model-1. Although this attention mechanism lacks ability to capture channel-wise importance in the extracted feature maps during processing, our proposed CNN with SCA model outperformed all other models.

**Figure 9:**
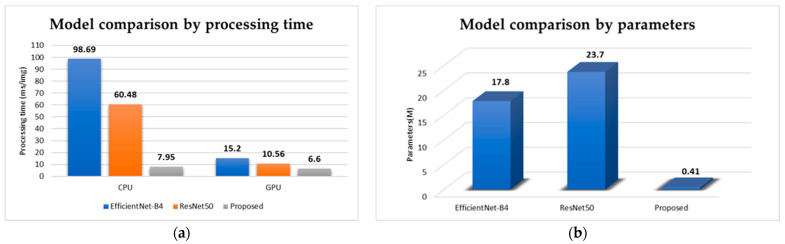
Illustration of the comparison of the proposed model performance in terms of processing time (**a**) and number of model parameters (**b**).

**Figure 10:**
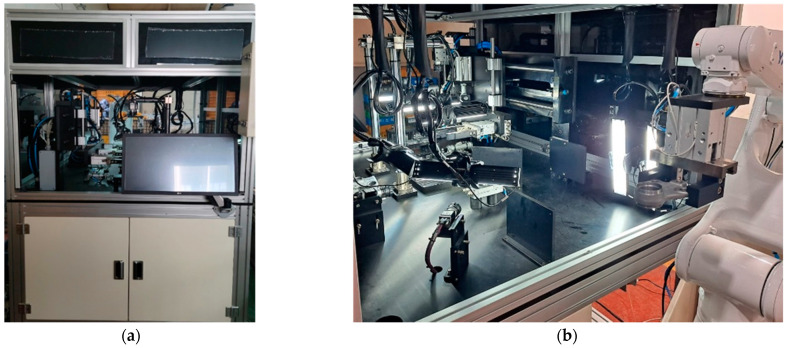
A demonstration of the 3D ball socket defect inspection system installed at the factory. (**a**) Outside view of the inspection system, (**b**) inside view of the inspection system.

We fine-tuned the pretrained Resnet50 [42] and EfficientNet-B4 [43] CNN models and tested them using the 3D ball joint test dataset. The results of the pretrained CNN models were 92% and 94%, respectively. We also compared processing time as shown in Figure 9a, and number of parameters of each model as shown in Figure 9b. The classification accuracy of both pretrained CNN models was over 90% for the 3D ball joint test dataset because these pretrained networks were well designed for the task of image classification and proved their efficiency in ImageNet [44] dataset. However, it is hard to apply them in real time inspection processes because of their large number of parameters and long processing time. Figure 10 shows the final defect inspection system deployed at the factory.

## 7. Conclusions

In this work, we developed and proposed a defect inspection system that uses a 2D convolutional neural network (CNN) with spatial and channel attention (SCA) mechanisms to efficiently classify 3D ball joint socket products into defect and good categories. In our developed system, five 2D industrial cameras were used to capture all parts of the 3D shape product in order to detect defects in a product. Gamma correction is applied to all captured images before feeding to the CNN with SCA. The main purpose of gamma correction is to fix the issue of light reflection from the product surface. During the development process of defect inspection system, we developed and experimented with four different CNN models to accurately classify defective and good quality products. The CNN model with SCA achieved 98% average testing accuracy rate which was the highest among other developed models. The rest of the models detected big and medium sized defect types with high accuracy, however, the recognition rate on small defect types was low. Additionally, in order to analyze proposed model performance, we compared model accuracy, parameters, and processing time with other attention mechanisms and pretrained CNN models. As a result of comparison attention mechanisms, such as squeeze-and-excitation block (SEB) and self-attention mechanism (Self-A), shows lower accuracy comparing to pretrained CNN models and the proposed CNN model with SCA. Although the pretrained CNN models achieved over 90% accuracy, it is hard to apply these models in fast real-time processing due to their large number of parameters and long processing time. Our proposed CNN model with SCA extracts useful features from the input image using a feature extraction block (FEB), and then channel attention detects the most relevant feature maps for the target while spatial attention finds the most important regions in the extracted feature map for the target, and then both attention features are combined to make a robust feature vector as well as detect defective products. Our proposed model detected defective products with 97% accuracy rate, while the recognition rate of good products was 99%.

In the future, we will collect more defective samples from the factory during the production and processing in our developed system. With efficient data samples, we will retrain and improve the recognition rate of our proposed model.

## Figures and Tables

**Table 1:** Detailed configuration specifications of our proposed model. Conv2D—Convolution2D, MP—MaxPooling2D, GAP—GlobalAveragePooling2D, ReLU—rectified linear unit, Con—concatenate, FEB—feature extraction block, SA—spatial attention, CA—channel attention, FCN—fully connected network, FC—fully connected layer.

Name of Layers	Input Tensor Shape	Output Tensor Shape	Kernel Size	Stride	Activation Function	Number of Parameters
FEB						
Conv2D_1	380 × 380 × 3	378 × 378 × 32	3 × 3	1 × 1	ReLU	896
MP_1	378 × 378 × 32	189 × 189 × 32	2 × 2	2 × 2	-	0
Conv2D_2	189 × 189 × 32	187 × 187 × 64	3 × 3	1 × 1	ReLU	18,496
MP_2	187 × 187 × 64	93 × 93 × 64	2 × 2	2 × 2	-	0
Conv2D_3	93 × 93 × 64	91 × 91 × 64	3 × 3	1 × 1	ReLU	36,928
MP_3	91 × 91 × 64	45 × 45 × 64	2 × 2	2 × 2	-	0
Conv2D_4	45 × 45 × 64	43 × 43 × 64	3 × 3	1 × 1	ReLU	36,928
MP_4	43 × 43 × 64	21 × 21 × 64	2 × 2	2 × 2	-	0
Conv2D_5	21 × 21 × 64	19 × 19 × 64	3 × 3	1 × 1	ReLU	36,928
MP_5	19 × 19 × 64	9 × 9 × 64	2 × 2	2 × 2	-	0
Conv2D_6	9 × 9 × 64	9 × 9 × 128	3 × 3	1 × 1	ReLU	73,856
MP_6	9 × 9 × 128	4 × 4 × 128	2 × 2	2 × 2	-	0
Conv2D_7	4 × 4 × 128	4 × 4 × 128	3 × 3	1 × 1	ReLU	147,584
MP_7	4 × 4 × 128	2 × 2 × 128	2 × 2	2 × 2	-	0
GAP_1	2 × 2 × 128	128	-	-	-	0
SA						
Conv2D_8	2 × 2 × 128	2 × 2 × 64	1 × 1	1 × 1	ReLU	8256
Conv2D_9	2 × 2 × 64	2 × 2 × 64	3 × 3	1 × 1	ReLU	36,928
Conv2D_10	2 × 2 × 64	2 × 2 × 64	1 × 1	1 × 1	ReLU	4160
GAP_2	2 × 2 × 64	64	-	-	-	0
CA						
GAP_3	2 × 2 × 128	128	-	-	-	0
FC_1	128	128	-	-	ReLU	16,512
FC_2	128	64			ReLU	8256
Con_1	128, 64, 64	256	-	-	-	0
FCN						
FC_3	256	128	-	-	ReLU	32,896
FC_4	128	64	-	-	ReLU	8256
FC_5	64	2	-	-	SoftMax	130
Total parameters = 467,010						

**Table 2:** Detailed description of the acquired 3D ball joint socket dataset. The dataset was divided into training and test sets with 80% and 20%, respectively. OK—non-defective products, NG—defective products.

Dataset Folders	Class Names and Number of Images	
	OK	NG
Train set	1014	734
Test set	255	150
Total	1269	884

**Table 3:** Environment specifications used to develop and test models.

Software Specifications	Hardware Specifications
OS: Windows 11 Pro	CPU: Intel Core i7-9700CPU @ 3.00 GHz RAM: 32 GB
Tools: TensorFlow 2.6, Python 3.8	GPU: NVIDIA GeForce RTX 3090

**Table 4:** A classification report of the proposed CNN model with SCA to demonstrate precision, recall, F1-score, and accuracy results. OK and NG indicate the non-defective and defective samples, respectively.

Class Category	Precision	Recall	F1-Score	Number of Images
OK	0.98	0.99	0.99	255
NG	0.98	0.97	0.98	150
**Accuracy**	**0.98**			

## Data Availability

Not applicable.
